# Effect of Intramuscular Protons, Lactate, and ATP on Muscle Hyperalgesia in Rats

**DOI:** 10.1371/journal.pone.0138576

**Published:** 2015-09-17

**Authors:** Nicholas S. Gregory, Phillip E. Whitley, Kathleen A. Sluka

**Affiliations:** 1 Neuroscience Graduate Program, University of Iowa, Iowa City, Iowa, United States of America; 2 Department of Physical Therapy and Rehabilitation Science, University of Iowa, Iowa City, Iowa, United States of America; 3 CFD Research Corporation, Huntsville, Alabama, United States of America; University of Texas Medical Branch, UNITED STATES

## Abstract

Chronic muscle pain is a significant health problem leading to disability[1]. Muscle fatigue can exacerbate muscle pain. Metabolites, including ATP, lactate, and protons, are released during fatiguing exercise and produce pain in humans. These substances directly activate purinergic (P2X) and acid sensing ion channels (ASICs) on muscle nociceptors, and when combined, produce a greater increase in neuron firing than when given alone. Whether the enhanced effect of combining protons, lactate, and ATP is the sum of individual effects (additive) or more than the sum of individual effects (synergistic) is unknown. Using a rat model of muscle nociceptive behavior, we tested each of these compounds individually over a range of physiologic and supra-physiologic concentrations. Further, we combined all three compounds in a series of dilutions and tested their effect on muscle nociceptive behavior. We also tested a non-hydrolyzable form of ATP (α,β-meATP) alone and in combination with lactate and acidic pH. Surprisingly, we found no dose-dependent effect on muscle nociceptive behavior for protons, lactate, or ATP when given alone. We similarly found no effect after application of each two-metabolite combination. Only pH 4 saline and α,β-meATP produced hyperalgesia when given alone. When all 3 substances were combined, however, ATP (2.4μm), lactate (10mM), and acidic pH (pH 6.0) produced an enhanced effect greater than the sum of the effects of the individual components, *i*.*e*. synergism. α,β me ATP (3nmol), on the other hand, showed no enhanced effects when combined with lactate (10mM) or acidic pH (pH 6.0), *i*.*e*. additive. These data suggest that combining fatigue metabolites in muscle produces a synergistic effect on muscle nociception.

## Introduction

Chronic muscle pain is a common condition contributing to disability in millions of people worldwide. The initiation of chronic muscle pain is poorly understood and likely multifactorial. Recent work indicates that fatiguing muscle contractions can trigger long lasting mechanical hyperalgesia [2]. Further, there is decreased pH and local antagonism of acid sensing ion channel 3 (ASIC3) in the muscle prior to fatiguing muscle contractions prevents the development hyperalgesia [3], indicating that fatigue byproducts may contribute to the development of muscle pain. However, decreases in pH alone are unlikely to be the cause of muscle pain, as single-injection of pH does not trigger widespread and long-lasting muscle pain. In human subjects, pain is produced acutely when acidic pH is infused into the muscle and recovers within minutes after the infusion is stopped [4]. In animals, a single injection of acidic pH produces a short-duration (hours) hyperalgesia [5–7]; however, fatiguing exercise when combined with decreases in pH produces long-lasting hyperalgesia [2,8,9]. Further, pH is rapidly buffered in the muscle (minutes)[5,10], and thus longer-term hyperalgesia is likely not reflective solely of decreases in pH.

Physiologic concentrations of protons, lactate, or ATP given individually trigger calcium influx in rat dorsal root ganglion (DRG) [11], and can produce pain when injected or infused into muscle in human subjects [4,12]. These 3 substances are particularly interesting because they may interact to enhance afferent activity and, subsequently, pain. In animals infusion of ATP at acidic pH in skin enhances hyperalgesia [7], and in human subjects injection of ATP, acidic pH and lactate into muscle in combination produces pain [11,12]. DRG neurons innervating muscle exposed to a combination of acid, lactate and ATP show enhanced calcium influx and ASIC current [11,13,14]. However, it is not known if this combination of ATP, lactate and protons produces enhanced hyperalgesia, and if this enhancement is synergistic or additive. We hypothesize that combining acidic pH, lactate and ATP would have a synergistic response to produce hyperalgesia and would be greater than the sum of the individual effects. Therefore, in the current study, we test the effects of pH, lactate, ATP, and the non-hydrolyzable α,β-methylene-ATP (α,β–meATP) alone and in combination on the withdrawal threshold of the muscle.

## Materials and Methods

These experiments were approved by the Animal Care and Use Committee at the University of Iowa. Male Sprague-Dawley rats (225-250g, Harlan, n = 287) were used for these studies.

### Muscle Withdrawal Threshold

To assess muscle hyperalgesia, muscle withdrawal thresholds (MWT) were measured by applying force sensitive tweezers to the belly of the gastrocnemius muscle as previously described [15], where lower thresholds indicate greater mechanical sensitivity. Rats were acclimated to a gardener’s glove in two five minute sessions per day over two days prior to behavioral testing. On the day of testing, rats were placed in a gardener’s glove, the hindlimb was held in extension, and the muscle was squeezed with force sensitive tweezers until the animal withdrew its hindlimb. The average of 3 trials per animal was recorded at each time period. A decrease in withdrawal thresholds was interpreted as muscle hyperalgesia. This measurement represents pressure pain thresholds and tenderness typically observed clinically in people with muscle pain [16].

### Drugs

Acidic saline (pH 4 to 7), lactate (Sigma-Aldrich, 1.5 M to 474 μM), ATP (Sigma-Aldrich, 760 nM to 24 mM), α,β–meATP (1 nM to 100 nM, Sigma-Aldrich), and 3% carrageenan (Sigma-Aldrich) solutions were prepared in 0.9% saline. For injections of ATP or lactate alone, pH was adjusted to pH 7.4 prior to injection. All drugs were prepared just prior to injection. A single 100 μL intramuscular injection was given to the gastrocnemius muscle while the rat was anesthetized with 4% isoflurane. Each rat only received a single injection.

### Experimental protocols

All behavior testing was done with the tester blinded to the substance injected. One person was responsible for random allocation of groups and preparation, blinding, and injection of drugs. A separate person was responsible for all behavior testing.

The first series of experiments tested the individual short-term effects of acidic saline, lactate, and ATP alone on muscle withdrawal threshold. In human nociceptors, electrophysiological recordings activate the channel within seconds and pain occurs immediately with application of ATP and is not long-lasting [12,17]. Similarly, protons and lactate, which activate ASICs, produce a pain rating when applied to muscle, but the effect is short-lived [4,12]. The duration of pain is longer-lasting when given in combination but again this all occurs within a short-duration [12]. Commonly behavioral studies examining the effects of receptor activation with endogenous ligands will test within 30 minutes, and previous studies show a decrease in withdrawal threshold 30 minutes after injection of pH 4.0 saline in mice or α,βmeATP in rats [6,7]. Therefore, initial experiments tested the effect of multiple doses of each drug across potential physiological ranges on muscle withdrawal threshold 30 minutes after injection.

For acidic saline, rats were injected with normal saline adjusted to neutral pH (n = 6), pH 4.0 (n = 12), pH 4.5 (n = 6), pH 5.0 (n = 6), or pH 6.0 (n = 6). For lactate, rats were injected with normal saline adjusted to pH 7.4 with lactate concentrations of 1.5 M (n = 6), 470 mM (n = 6), 150 mM (n = 6), 47 mM (n = 12), 15 mM (n = 12), 4.7 mM (n = 6), 1.5 mM (n = 6), and 470 μM (n = 6), or saline control (n = 6). For ATP, rats were injected with normal saline adjusted to pH 7.4 with ATP concentrations of 24 mM (n = 6), 7.6 mM (n = 6), 2.4 mM (n = 6), 760 μM (n = 12), 76 μM (n = 6), 760 nM (n = 6), 7.6 μM (n = 6), or 760 nM (n = 6).

Since ATP was ineffective, is quickly degraded, and a prior study shows hyperalgesia after injection of α,β–me ATP into muscle within 30 minutes [18,19], we tested for dose-response effects of α,β–meATP at 30 minutes after injection at concentrations of 100 nM (n = 5), 30 nM (n = 5), 10 nM (n = 4), 3 nM (n = 4), 1 nM (n = 4), and saline control (n = 7).

The second series of experiments then evaluated the effects of combining ATP or α,β–me ATP with lactate and protons on muscle withdrawal threshold 30 minutes after injection. We used a series of isobole solutions across multiple dose ranges. See Tables 1 and 2 for doses and sample sizes of the ATP and α,β–meATP groups, respectively.

**Table 1:** Doses Used in the Acidic pH, Lactate, ATP Isobole Dose Response Curve (Fig 2A).

Group	pH	Lactate	ATP
Combination 1	6.0	474 μM	2.4 μM
Combination 2	6.5	150 μM	760 nM
Combination 3	7.0	47.4 μM	240 nM
Combination 4	7.5	15 μM	76 nM

**Table 2:** Doses Used in the Acidic pH, Lactate, αβ–meATP Isobole Dose Response Curve (Fig 2B).

Group	pH	Lactate	α,β–me ATP
Combination 1	6.0	474 μM	3 nmol
Combination 2	6.4	150 μM	1 nmol
Combination 3	7.0	47 μM	0.3 nmol
Combination 4	7.5	15 μM	0.1 nmol
Combination 5	6.0	10 mM	3 nmol

The third series of experiments tested if a longer-duration effect occurred during the synergism which could occur through intracellular communications between channels as proposed in prior studies [14]. In fact, a more recent animal study showed delayed effects of the combination of α,β–meATP with acidic pH (pH 4.0) when applied subcutaneously [7]. Therefore, we tested the muscle withdrawal thresholds over a 4-hour time period after combining acidic pH (pH 6.0), lactate and either ATP or α,β–me ATP. We tested the combined effect of all three metabolites and compared this to each compound alone to examine for synergy. Concentrations were derived from the above experiments that had no significant effect and were within the physiological range. Specifically, for the ATP study muscle withdrawal threshold was measured after intramuscular injection with an injection of a single substance: pH 6 saline (n = 4), 10 mM lactate (n = 4), 2.4 μM ATP (n = 4). Muscle withdrawal threshold was then tested in each possible pairwise combination (2.4 μm ATP + pH 6, n = 4; 2.4 μm ATP + 10 mM lactate, n = 4; pH 6.0 + 10 mM lactate, n = 4) at baseline and 30 minutes, 1 hour, 2 hours, and 4 hours after injection of the combined solution. Lastly, muscle withdrawal thresholds were tested with all 3 substances in combination (2.4 μm ATP, pH 6 and 10 mM lactate, n = 5). For the α,β–me ATP study, muscle withdrawal threshold was measured after intramuscular injection with pH 6 saline (n = 4), 10 mM lactate (n = 4), 3 nM α,β–meATP (n = 4), or all three in combination (n = 6).

## Results

### Effects of acidic pH, ATP and lactate on muscle withdrawal threshold

To test if single injections of acidic pH, ATP or lactate modulate muscle withdrawal thresholds, we performed a dose-response analysis for effects on muscle withdrawal threshold for each substance alone. Muscle withdrawal thresholds were assessed at baseline and 30 minutes after injection of the drug.

#### pH 4 saline produces significant decrease in muscle withdrawal thresholds

**Fig 1:**
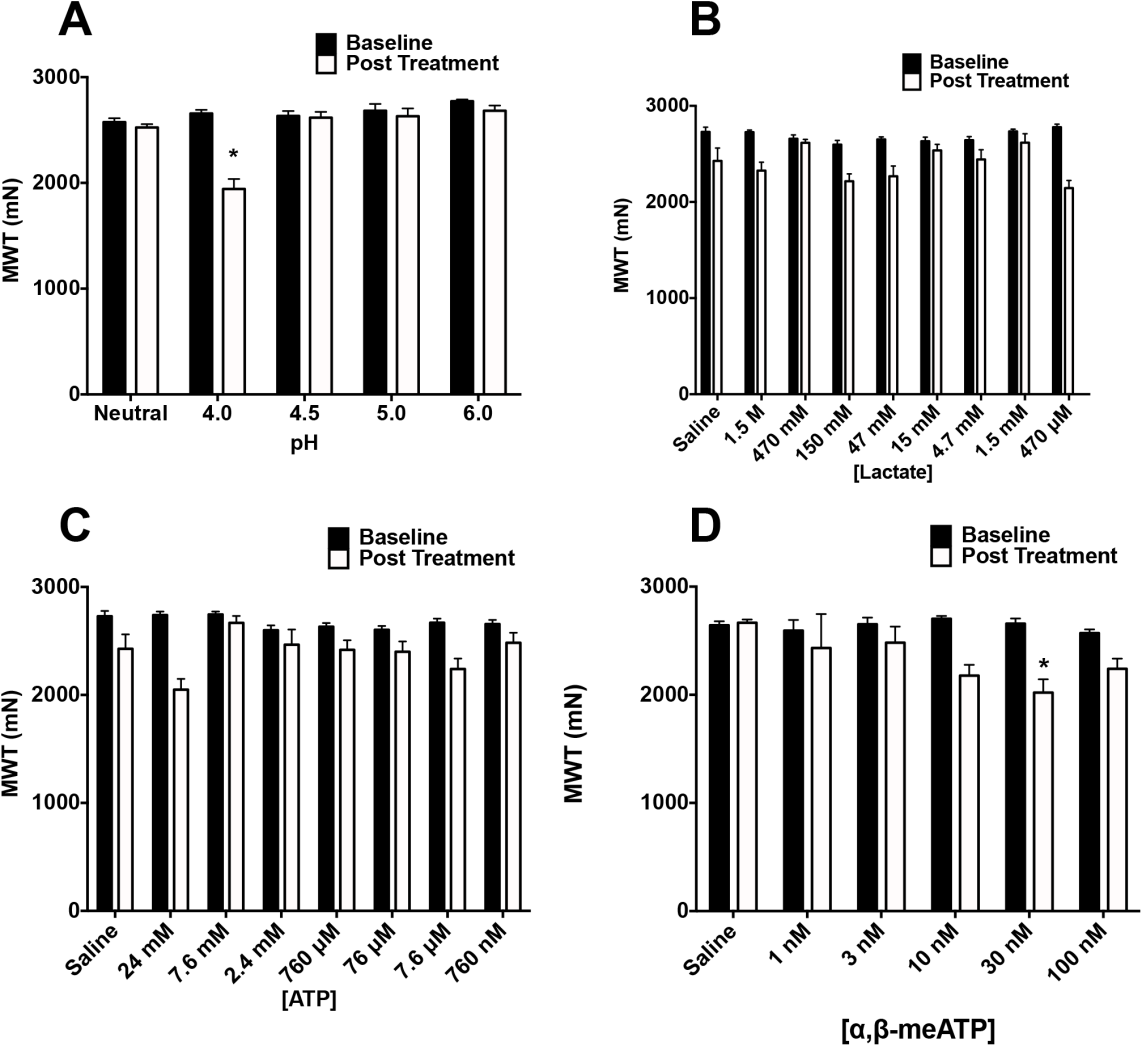
Effect of Intramuscular Injection of pH, lactate, ATP, and α,β–meATP on Muscle Withdrawal Threshold. Muscle withdrawal threshold was measured before and 30 minutes after injection of physiologic and supraphysiologic concentrations of pH, lactate, ATP, and α,β–meATP. (A) pH dose response curve. Rats were injected with normal saline adjusted to neutral pH (n = 6), pH 4.0 (n = 12), pH 4.5 (n = 6), pH 5.0 (n = 6), and pH 6.0 (n = 6). Of these doses, only pH 4.0 saline produced a significant decrease in muscle withdrawal threshold from baseline (Repeated measures ANOVA, F_4,31_ = 16.761, p <0.001, Tukey test, p < 0.001). (B) Lactate dose response curve. Rats were injected with normal saline adjusted to pH 7.4 with lactate concentrations of 1.5 M (n = 6), 470 mM (n = 6), 150 mM (n = 6), 47 mM (n = 12), 15 mM (n = 12), 4.7 mM (n = 6), 1.5 mM (n = 6), and 470 μM (n = 6), saline control (n = 6). There was no significant change from baseline as compared to saline control (Repeated measures ANOVA, F_7,52_ = 1.834, p = 0.100). (C) ATP dose response curve. Rats were injected with normal saline adjusted to pH 7.4 with ATP concentrations of 24 mM (n = 6), 7.6 mM (n = 6), 2.4 mM (n = 6), 760 μM (n = 12), 76 μM (n = 6), 760 nM (n = 6), 7.6 μM (n = 6), 760 nM (n = 6). ATP concentration had a significant effect on muscle withdrawal threshold (Repeated measures ANOVA, F_7,46_ = 2.315, p = 0.041), but there was no significant difference between any dose and the saline control (Tukey test, p > 0.05 for all doses versus saline control). (D) α,β–me ATP dose response curve. Rats were injected with normal saline adjusted to pH 7.4 with α,β–meATP concentrations of 100 nM (n = 5), 30 nM (n = 5), 10 nM (n = 4), 3 nM (n = 4), 1 nM (n = 4), and saline control (n = 7). α,β–meATP significantly decreased muscle withdrawal threshold (Repeated measures ANOVA, F_5,23_ = 6.949, p < 0.001), with the 100 nM and 30 nM doses being effective (Tukey test, p < 0.05 each dose). *, p < 0.05.

The effect of intramuscular injection of protons (pH) on muscle withdrawal thresholds was tested using isotonic saline across a range of pH values (pH 4.0–7.4). pH had a significant effect on muscle withdrawal threshold 30 minutes after injection (Fig 1A, repeated measures ANOVA, F_4,31_ = 16.761, p <0.001). Of the pH values injected intramuscularly, only pH 4 saline produced a significant decrease in the muscle withdrawal threshold 30 minutes after injection relative to saline injected controls (Tukey test, p < 0.001).

#### Lactate alone does not decrease muscle withdrawal threshold

The effect of intramuscular injection of lactate on muscle withdrawal threshold was tested using a range of concentrations, both in the physiologic and supra-physiologic ranges (450μM to 1.5 M). Lactate injected intramuscularly had no significant effect on muscle withdrawal thresholds over this range of doses (Fig 1B, repeated measures ANOVA, F_7,52_ = 1.834, p = 0.100).

#### ATP alone does not decrease muscle withdrawal threshold

The effect of intramuscular injection of ATP on muscle withdrawal threshold was tested using a number of concentrations (24 mM to 760 nM). ATP had a significant effect on muscle withdrawal threshold (Fig 1C, repeated measures ANOVA, F_7,46_ = 2.315, p = 0.041), but no doses were significantly different from the saline control (Tukey test, p > 0.05).

#### α,β-methylene ATP reduces muscle withdrawal threshold

Since ATP alone had no effect, ATP is quickly metabolized in muscle, and prior studies have shown hyperalgesia after injection of the non-hydrolyzable ATP analog α,β–me ATP [18,19], we performed a dose-response analysis after injection of α,β–me ATP. α,β–meATP had a significant effect on muscle withdrawal threshold (Fig 1D, repeated measures ANOVA, F_5,23_ = 6.949, p < 0.001). The two highest doses (30 nM & 100 nM) significantly reduced the muscle withdrawal threshold as compared to pH 7.2 saline (Tukey test, p < 0.05 for each dose), while the 10 nM dose was nearly significant (Tukey test, p = 0.062).

### Effect of combinations of protons, lactate, and ATP on muscle withdrawal threshold

#### Combining protons, lactate, and ATP reduces muscle withdrawal threshold

**Fig 2:**
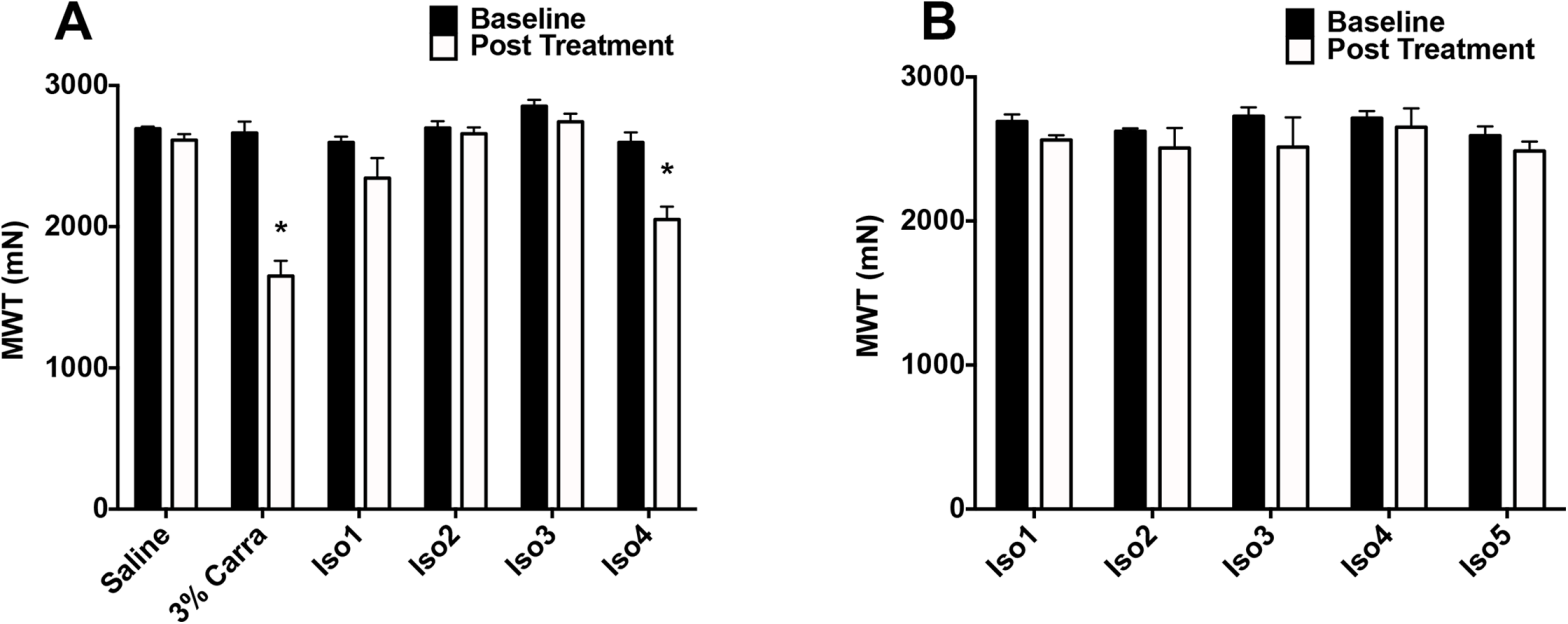
Combination of ATP (A) or αβ–meATP (B) with Lactate and Protons. Muscle withdrawal thresholds were assessed before and 30 min after intramuscular injection. (A) Protons, lactate, and ATP were given in combination across series dilutions. Combining protons, lactate, and ATP had a significant effect on muscle withdrawal (Repeated measures ANOVA F_5,27_ = 17.986, p < 0.001), but only the most dilute combination (C4) was decreased relative to saline controls (Tukey test, p = 0.001). The positive control, intramuscular injection of 3% carrageenan shows a significant decrease 24h after injection (p<0.001). (B) Protons, lactate, and α,β–meATP were given in combination across a series of dilutions. None of combinations tested produced significant decreases in muscle withdrawal threshold (Repeated-measures ANOVA F_3,14_ = 5.373, p < 0.011). *, p < 0.05.

To test for synergism between the agonists we combined 3 doses in the lowest physiological range that were ineffective; development of hyperalgesia would indicate synergism [20,21]. We then injected half-log dilutions of this fixed ratio into the gastrocnemius muscle of rats and measured muscle withdrawal thresholds 30 minutes after injection (Table 1). The concentration of this combination had a significant effect on muscle withdrawal threshold (Fig 2A, repeated measures ANOVA, F_5,27_ = 17.986, p < 0.001). Surprisingly, only the most dilute combination significantly decreased with muscle withdrawal threshold (post-hoc Tukey test, p = 0.001). When α,β–me ATP in combination with lactate (10 mM to 15uM) and acidic pH (6.0 to 7.5) across several fixed dilutions (Table 2), there was no significant decrease in muscle withdrawal threshold at the 30 minute time point (repeated-measures ANOVA F_4,17_ = 0.637, p = 0.643). A positive control, 3% carrageenan injected into the muscle, showed a significant decrease in withdrawal threshold in this group (Fig 2).

#### Effect of combining components versus each component alone

**Fig 3:**
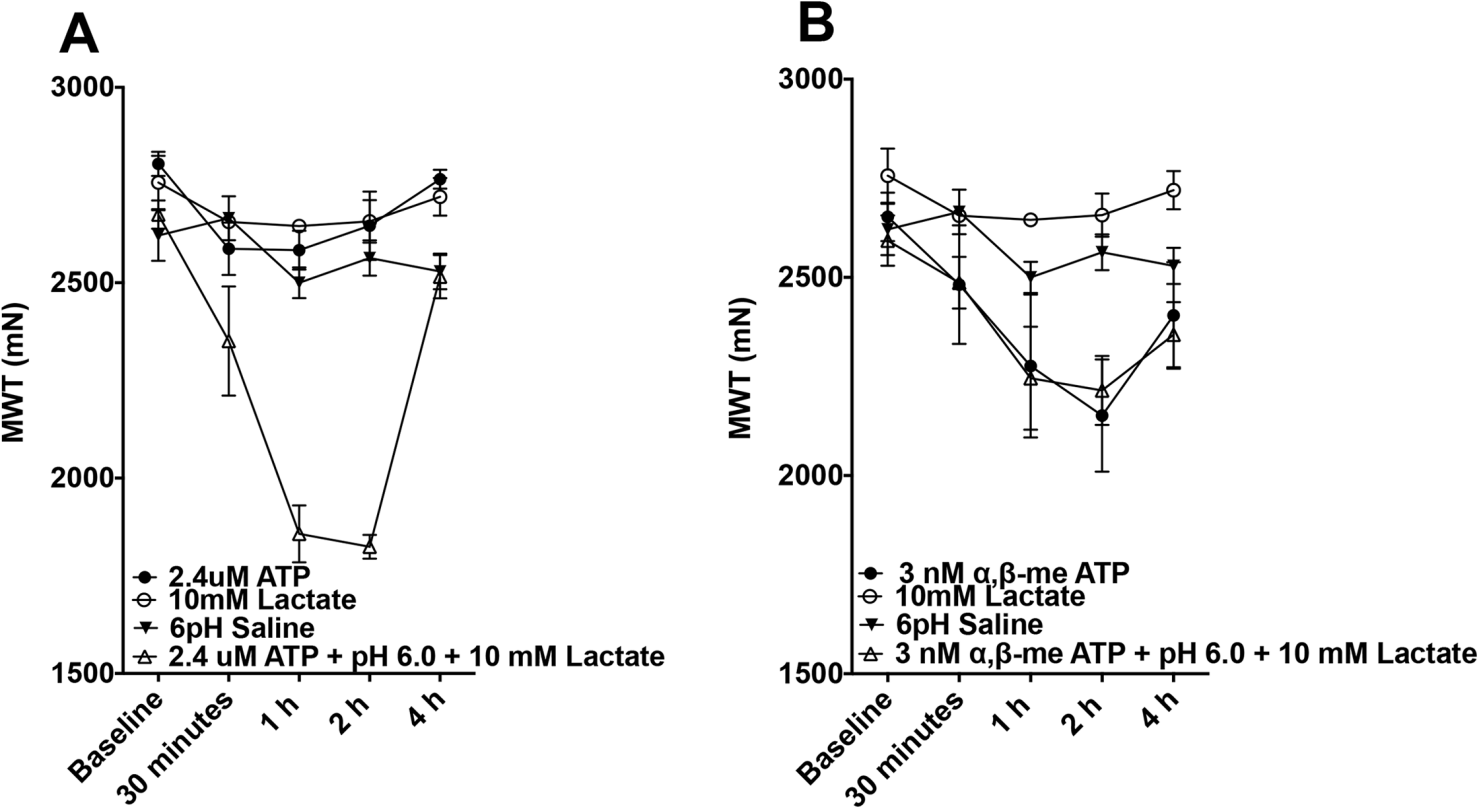
Comparison of Individual Components Versus Combined Solution on muscle withdrawal thresholds across a 4h time period. (A) Injection of protons, lactate, and ATP alone compared to the combined solution. Muscle withdrawal threshold was measured before and after intramuscular injection with pH 6 saline (n = 4), 10 mM lactate (n = 4), 2.4 μM ATP (n = 4), and all three in combination (n = 5). None of the individual components had an effect on muscle withdrawal threshold, but the combination produced significant decreases (Repeated measures ANOVA, F_3,13_ = 54.568, p < 0.001, Tukey test, p < 0.05 for each compound alone). (B) Injection of protons, lactate, and α,β–meATP alone compared to the combined solution. Muscle withdrawal threshold was measured after intramuscular injection with pH 6 saline (n = 4), 10 mM lactate (n = 4), 3 nM α,β–meATP (n = 4), or all three in combination (n = 6). The 3 nM α,β–meATP and the combination solution produced similar decreases in muscle withdrawal threshold (Repeated-measures ANOVA F_3,14_ = 5.373, p < 0.011, Tukey test p > 0.05 for 3 nM α,β–meATP compared to combination solutions, p < 0.05 the remaining) *, p < 0.05.

**Fig 4:**
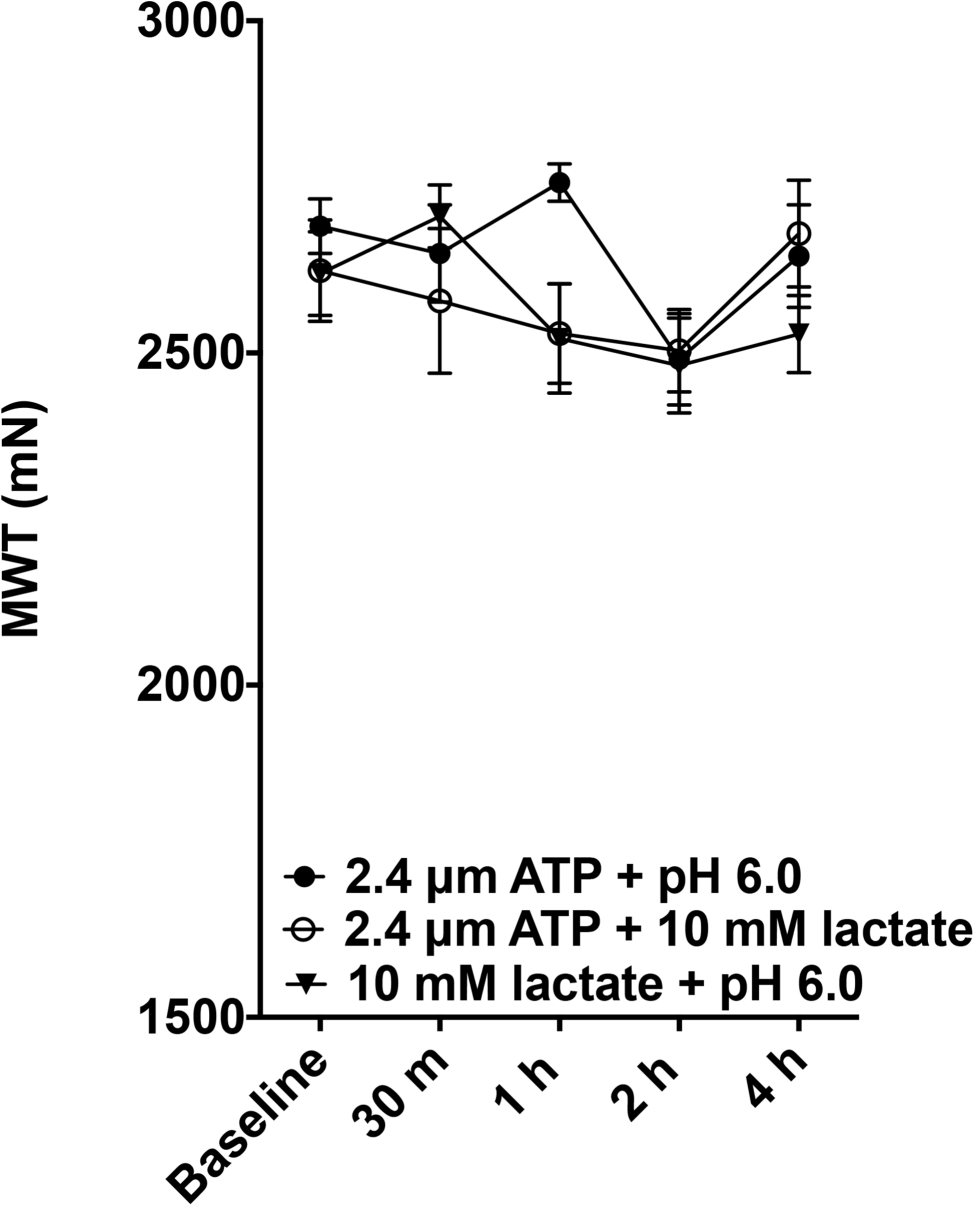
Effects of paired solutions on muscle withdrawal thresholds across a 4h time period. Injection of the dual combinations of metabolites. Muscle withdrawal threshold were measured before and after each possible pairwise combination (2.4 μm ATP + pH 6, n = 4; 2.4 μm ATP + 10 mM lactate, n = 4; pH 6.0 + 10 mM lactate, n = 4). There was no significant difference for the paired combinations (repeated measures ANOVA, F_2,9_ = 0.911, p = 0.436).

We repeated the combined solutions using a higher dose of lactate used in a recent publication studying human subjects [12] and followed the pain behavior out to 4 hours. Additionally, we compared the effect of combined doses of these substances when given in paired combinations, or when all 3 substances were given together to the effect of the individual components to test for synergism. A significant decrease in muscle withdrawal thresholds was observed when pH 6.0, 2.4 μM ATP, and 10 mM lactate were combined when compared to pH 6.0 saline, 2.4 μM ATP, or 10 mM lactate alone (Fig 3A, repeated measures ANOVA, F_3,13_ = 54.568, p < 0.001, Tukey test, p < 0.05 for each compound alone). There were no significant decreases in withdrawal threshold for these doses of fatigue metabolites (acid, ATP, lactate) when given individually, or for any of the paired combinations (Fig 4; repeated measures ANOVA, F_2,9_ = 0.911, p = 0.436).

In contrast, while the combined dose of 3 nM α,β–meATP, pH 6.0 and 10 mM did produce significant decreases in muscle withdrawal threshold from baseline (Fig 3B, repeated-measures ANOVA F_3,14_ = 5.373, p < 0.011), it was not significantly different from 3 nM α,β–meATP alone (Tukey test, p > 0.05).

## Discussion

The current study shows that intramuscular injection of either acidic saline or the ATP analogue α,β–meATP is sufficient to produce mechanical hyperalgesia in the gastrocnemius muscle of rats; however, ATP or lactate given alone produce no hyperalgesia. On the other hand, combining protons, lactate, and ATP at concentrations that have no effect on their own produces hyperalgesia, thus showing synergy between the 3 fatigue metabolites. This synergy does not develop with α,β–me ATP. These data reinforce the notion that acid, lactate, and ATP produce a synergistic effect on muscle nociceptors to induce hyperalgesia.

### Acidic pH and Pain

Previous studies show low pH produces pain in humans [4,22] and animals [5,23–25]. In humans, intramuscular infusion of buffered pH 5.2 saline produces pain and hyperalgesia both local to the muscle and distantly at the ankle [4,22]. In mice, a single injection of acid in the skin or muscle produces a short duration mechanical hyperalgesia [5,23–25] that depends on activation of ASIC3 [6,24]. The current study extends these findings by showing muscle withdrawal thresholds decreases after pH 4.0 saline, and the hyperalgesia is eliminated by increasing pH 0.5 units (pH 4.5). Intramuscular pH likely does not decrease to the value of the injected solutions because of the buffering capacity of the muscle and clearance from the tissue. In fact, intramuscular injection of pH 4.0 reduces the pH in muscle to an average of pH 6.5 for less than 10 minutes in rats [5].

Decreases in pH occur in multiple conditions including inflammation, incision, and exercise [26–30]. For example, incision of the gastrocnemius muscle in animals decreases pH to 6.76 [30], and enhances the response of DRGs [31]. Exercise and muscle fatigue decrease muscle pH to similar levels in humans and animals [3,28,29]. Yet, pH 6.6 solutions applied to rat DRG innervating muscle do not trigger calcium influx [11] nor is infusion of pH 6.6 saline reported as painful by human subjects [12].These data suggest that pH has a narrow range over which it produces hyperalgesia, the decrease requires ASIC3 for induction of hyperalgesia, and other factors work with acidic pH to induce hyperalgesia.

### Lactate and Pain

In humans, lactate is normally present in interstitial fluid at approximately 1mM and can increase to 10mM after fatiguing exercise [32], but lactate alone does not produce pain [12]. In rats, muscle incision increases tissue lactate concentration (3.6–4.2 mM) and produces hyperalgesia [33,34]. Lactate alone at a normal pH minimally increases intracellular calcium concentration in isolated rat DRG neurons [11], but when pH is decreased the effects of lactate are potentiated [11]. In cell culture, lactate acts as a Ca2+-chelator and potentiates the response of ASICs to protons by facilitating the displacement of Ca2+ from the acidic pocket of ASICs to increase channel opening [13,35]. In the present study, we show lactate by itself is not sufficient to produce hyperalgesia, suggesting that even at high concentrations, protons are needed to displace the Ca2+ bound to the acidic pocket and open ASICs.

### ATP and Pain

In humans, injection of low dose ATP (5μM) into the hypothenar muscle is not painful [12], but injection of higher doses (9–36 μM) into the trapezius muscle produces spontaneous pain and hyperalgesia [36]. Injection of ATP into the muscle activates muscle nociceptors in animals [37]. ATP binds to a range of both metabotropic (P2Y) and ionotropic (P2X) receptors [38], and thus could produce effects at multiple targets. P2X receptors are expressed on nociceptive neurons. In the current study, however, we show no muscle hyperalgesia despite using a wide range of ATP concentrations (760 nM to 24 mM), including those well above an effective dose in humans [36]. The basis for this difference is unclear but may represent differences in metabolism, volume of injection, or the muscle injected.

In contrast, we show injection of a non-hydrolyzable form of ATP, α,β–meATP, does produce hyperalgesia. This is consistent with animal studies showing α,β–me ATP activates muscle nociceptors [39] and produces mechanical hyperalgesia [40] at similar concentrations used in the present study. Since α,β–meATP is more stable than ATP, it is possible that ATP was degraded rapidly and thus did not sufficiently activate purinergic receptors. Alternatively, α,β–meATP has a higher binding affinity to P2X1 and P2X3 [41] than ATP and thus activation of specific receptor subtypes could contribute to the hyperalgesia.

### Synergism between pH, lactate and ATP

Physiologic combinations of pH, lactate, and ATP activate DRG neurons and produce pain in humans [11,12]. In the present study, we extend these findings by showing this low dose combination of (pH 7.5, 15 μM lactate, 76 nM ATP) produces muscle hyperalgesia and is synergistic. It is curious that the lowest concentration combination produced mechanical hyperalgesia. This may mean that increasing concentration of these compounds is not sufficient to produce hyperalgesia; rather, concentrations must be within a specific range for the receptors to be activated. In subsequent experiments, we used a higher dose of lactate with physiological doses of ATP and pH (pH 6.0, 10 mM lactate, 2.4 μM ATP), doses similar to that used in humans [12]. We show the interaction between these 3 substances is synergistic, and that their effects are long-lasting. Lower concentrations of these compounds (pH 7.3, 400 nM ATP, 5 mM lactate) injected into the muscle produce warmth and fatigue sensations, while pain is reported with injection of higher concentrations of the combined compounds in humans (pH 7.2, 500 nM ATP, 10 mM lactate—pH 6.6, 5 μM ATP, 50 mM lactate) [12]. The fact that three ineffective doses when combined together cause significant decreases in muscle withdrawal threshold suggests protons, lactate, and ATP act synergistically to produce mechanical hyperalgesia [20,21]. Further, we show combining all 3 substances is necessary to produce the mechanical hyperalgesia, as each paired combination failed to produce mechanical hyperalgesia. This is consistent with previous studies showing acid-evoked currents and calcium influx in muscle DRG are potentiated, and the greatest effects occur, by combining all 3 metabolites [11,13,14]. The present behavior studies also show a slow onset requiring 1–2 hours for maximal hyperalgesia. This hyperalgesia lasts for hours after a single injection, suggesting activation of cellular processes which are independent of ion channel effects, activation of other cell types such as macrophages [3], and/or triggering release of inflammatory cytokines [19].

Surprisingly, no synergism was observed with α,β–meATP in combination with lactate and acidic pH in the current study. This behavioral result parallels the observation that α,β–me ATP does not potentiate acid-evoked currents in studies of cultured DRGs [14], but differs from prior behavioral studies showing potentiation when combined with protons [7]. α,β–me ATP has a higher binding affinity to P2X1 and P2X3 [41], and selective P2X1 and P2X3 antagonists fail to block calcium influx in DRG neurons triggered by protons, lactate, and ATP or acid-evoked current after application of ATP [11,14]. Thus, the lack of synergistic effect could be related to the purinergic receptor activated by α,β-meATP, and suggests that P2X1 and/or P2X3 are not involved in the synergism observed with ATP. Thus, effects of ATP could be mediated through other P2X receptor or P2Y receptors [38]. In support, prior studies show acid-evoked currents and calcium influx in DRG is blocked by non-selective P2X antagonists [11,14], and downregulation or blockade of P2Y1 in peripheral afferents reduces nociceptive behaviors in an animal models of pain [42–44]

Several channels respond to lactate and protons including acid sensing ion channels (ASICs) ASIC1 and ASIC3, as well as TRPV1 [45–47]. We speculate that ASIC3 mediates the synergism between ATP, protons and lactate since ASIC3 is activated by pH over the range measured in painful muscle conditions [45], shows enhanced sensitivity by lactate [13], and forms a physical interaction with P2X channels [14]. Prior studies also show ASIC3 is expressed in muscle afferents [48], and is critical for the development of muscle pain due to acidic saline injection, inflammation, and muscle fatigue [3,6,49,50].

### Summary

In summary, the current study shows that acidic saline or α,β–me ATP, but not lactate or ATP alone, is sufficient to produce mechanical hyperalgesia in rats. When combined, protons, lactate, and ATP produce mechanical hyperalgesia at low concentrations, but have no effect at higher concentrations. This indicates that these compounds have a synergistic interaction, but that development of mechanical hyperalgesia develops within a physiologically relevant range for which receptors like ASICs and P2Xs are sensitive.
